# Supplementary material for: Convex-hull mass estimates of the dodo (Raphus cucullatus): application of a CT-based mass estimation technique
Source: PeerJ. 2016 Jan 11;4:e1432. doi: 10.7717/peerj.1432 (PMC4715441; doi:10.7717/peerj.1432)
Supplement: Supplemental Information 1 [file peerj-04-1432-s001.docx]

**Supplementary Material**

**Convex-hull based volumetric mass estimates of the dodo (*Raphus cucullatus)***

Brassey, C.A.^1^*, O’Mahoney, T.^1^, Kitchener, A.C.^2,3^, Manning, P. L.^4,5^, Sellers, W.I.^1^

^1^Faculty of Life Sciences, University of Manchester, Manchester M13 9PL

^2^Department of Natural Sciences, National Museums Scotland, Edinburgh EH1 1JF

^3^ Institute of Geography, School of Geosciences, University of Edinburgh, Drummond Street, Edinburgh, EH8 9XP

^4^Interdisciplinary Centre for Ancient Life, School of Earth, Atmospheric and Environmental Sciences, University of Manchester, Manchester M13 9PL

^5^ Department of Geology and Environmental Geosciences, College of Charleston, Charleston, South Carolina, United States of America

*corresponding author ([charlotte.brassey@manchester.ac.uk](mailto:charlotte.brassey@manchester.ac.uk))

**Supplementary Material S1.** Raw convex hull volume data for extant pigeon dataset. Volume in mm^3^.

|  | **Trunk** | **Skull** | **Neck** | **RFem** | **RTbt** | **RTmt** | **RFoot** | **RHum** | **RRad** | **Rcmc** |
| --- | --- | --- | --- | --- | --- | --- | --- | --- | --- | --- |
| *Goura victoria* | 1090865 | 50856 | 16529 | 15072 | 35558 | 7182 | 26394 | 34486 | 27520 | 19456 |
| *Streptopelia decaocto** | 142130 | 13967 | 3400 | 1505 | 2866 | 644 | 3672 | 4944 | 5307 | 3555 |
| *Columba livia* | 82278 | 9627 | 1438 | 980 | 1676 | 281 | 1484 | 2413 | 2222 | 2275 |
| *Columba palumbus** | 246696 | 19861 | 5185 | 2626 | 5806 | 799 | 4686 | 6890 | 6542 | 5416 |
| *Ducula bicolor* | 245809 | 21472 | 7275 | 2495 | 5306 | 974 | 4923 | 7712 | 5772 | 4782 |
| *Petrophassa rufipennis* | 197999 | 22322 | 7440 | 3072 | 6074 | 1303 | 8562 | 7741 | 6446 | 4523 |
| *Otidiphaps nobilis* | 253579 | 15875 | 5521 | 4205 | 10353 | 1793 | 9403 | 4314 | 3505 | 2286 |
| *Nesoenas mayeri* | 135927 | 11378 | 4588 | 1843 | 3731 | 631 | 5385 | 4201 | 3208 | 3076 |
| *Ducula* sp*.* | 229009 | 19190 | 7010 | 2487 | 4692 | 700 | 4714 | 6693 | 5793 | 4489 |
| *Caloenas nicobarica* | 257958 | 20513 | 5398 | 4235 | 8763 | 1629 | 7959 | 10640 | 9006 | 7674 |
| *Phaps chalcoptera* | 154885 | 12942 | 2867 | 1926 | 3762 | 602 | 2447 | 5354 | 4014 | 3385 |
| *Ducula aenea* | 250110 | 24164 | 5620 | 3048 | 5974 | 1290 | 5390 | 7753 | 5943 | 4865 |
| *Columba guinea* | 65703 | 10968 | 1881 | 1019 | 2129 | 335 | 1769 | 3370 | 2537 | 2705 |
| *Zenaida graysoni* | 75854 | 7273 | 2044 | 808 | 1841 | 476 | 1789 | 2076 | 1539 | 1512 |
| *Gallicolumba* sp. | 115073 | 9750 | 1982 | 1496 | 3484 | 836 | 5639 | 2712 | 2450 | 2120 |
| *Phapitreron leucotis* | 49645 | 6930 | 981 | 634 | 1098 | 258 | 1260 | 1083 | 1011 | 728 |
| *Ptilinopus* sp*.* | 32488 | 5622 | 803 | 371 | 866 | 256 | 621 | 1014 | 821 | 548 |
| *Ptilinopus superbus* | 53345 | 8087 | 1370 | 641 | 1200 | 238 | 1333 | 1644 | 1203 | 1015 |
| [*Treron*](http://en.wikipedia.org/wiki/Treron)*vernans* | 72766 | 9588 | 2150 | 861 | 1639 | 280 | 1538 | 2235 | 2075 | 1641 |
| *Ocyphaps lophotes* | 42257 | 7159 | 1110 | 693 | 1549 | 351 | 1974 | 1384 | 1178 | 1570 |

**Supplementary Material S1. (cont.)**

|  | **LFem** | **LTbt** | **LTmt** | **LFoot** | **LHum** | **LRad** | **Lcmc** |
| --- | --- | --- | --- | --- | --- | --- | --- |
| *Goura victoria* | 14881 | 35955 | 8036 | 24009 | 33405 | 28321 | 18647 |
| *Streptopelia decaocto* | 1473 | 2879 | 629 | 3412 | 5127 | 4157 | 4201 |
| *Columba livia* | 848 | 1683 | 301 | 1387 | 2739 | 2212 | 2092 |
| *Columba palumbus* | 2460 | 5221 | 991 | 5027 | 7194 | 6522 | 6061 |
| *Ducula bicolor* | 2654 | 4810 | 1277 | 3233 | 7904 | 5821 | 5148 |
| *Petrophassa rufipennis* | 3128 | 1375 | 5948 | 8844 | 8153 | 6160 | 4414 |
| *Otidiphaps nobilis* | 5042 | 10125 | 2149 | 5726 | 4321 | 3422 | 2739 |
| *Nesoenas mayeri* | 2074 | 3943 | 660 | 5804 | 4497 | 3525 | 2690 |
| *Ducula* sp*.* | 2497 | 4871 | 724 | 4927 | 6685 | 5514 | 4982 |
| *Caloenas nicobarica* | 4293 | 8664 | 1632 | 8024 | 9974 | 9107 | 8260 |
| *Phaps chalcoptera* | 1719 | 3901 | 577 | 2564 | 5315 | 4479 | 3206 |
| *Ducula aenea* | 2859 | 5482 | 1031 | 5909 | 7818 | 6304 | 4699 |
| *Columba guinea* | 1017 | 1947 | 330 | 1346 | 3287 | 2241 | 2566 |
| *Zenaida graysoni* | 930 | 1862 | 416 | 1546 | 2076 | 1539 | 1512 |
| *Gallicolumba* sp*.* | 1682 | 3375 | 768 | 5988 | 2749 | 2264 | 2156 |
| *Phapitreron leucotis* | 511 | 1151 | 286 | 1076 | 1086 | 947 | 731 |
| *Ptilinopus* sp*.* | 393 | 872 | 294 | 560 | 831 | 853 | 596 |
| *Ptilinopus superbus* | 677 | 1249 | 180 | 1858 | 1578 | 1187 | 1070 |
| *Treron vernans* | 780 | 1704 | 301 | 1468 | 2126 | 2010 | 1822 |
| *Ocyphaps lophotes* | 726 | 1519 | 300 | 1466 | * | 1183 | 1588 |

*humerus badly damaged, right humerus mirrored instead

**Supplementary Material S2.** Results of phylogenetic generalised least squared (PGLS) regression analyses.

| Model | *a* | *a* (±95%) | *b* | *b* (±95%) | *r*^2^ | MSE | AIC |
| --- | --- | --- | --- | --- | --- | --- | --- |
| Eviscerated | -2.69 | -3.19- -2.34 | 0.96 | 0.89-1.05 | 0.97 | 0.009 | -20.97 |
| - minus feet | -2.67 | -3.14- -2.30 | 0.96 | 0.90-1.05 | 0.97 | 0.008 | -22.22 |
| Intact | -0.75 | -1.32- 0.04 | 0.60 | 0.45-0.70 | 0.91 | 0.008 | -10.15 |
| - minus feet | -0.72 | -1.30- 0.01 | 0.59 | 0.45-0.70 | 0.91 | 0.008 | -10.41 |
| Combined | -2.53 | -3.02- -1.97 | 0.93 | 0.84-1.03 | 0.94 | 0.013 | -26.64 |
| - minus feet | -2.51 | -3.13- -2.00 | 0.93 | 0.84-1.04 | 0.94 | 0.012 | -27.39 |

**Supplementary Material S2 continued**. Phylogeny used in PGLS analysis. Derived from Jetz et al. [28]


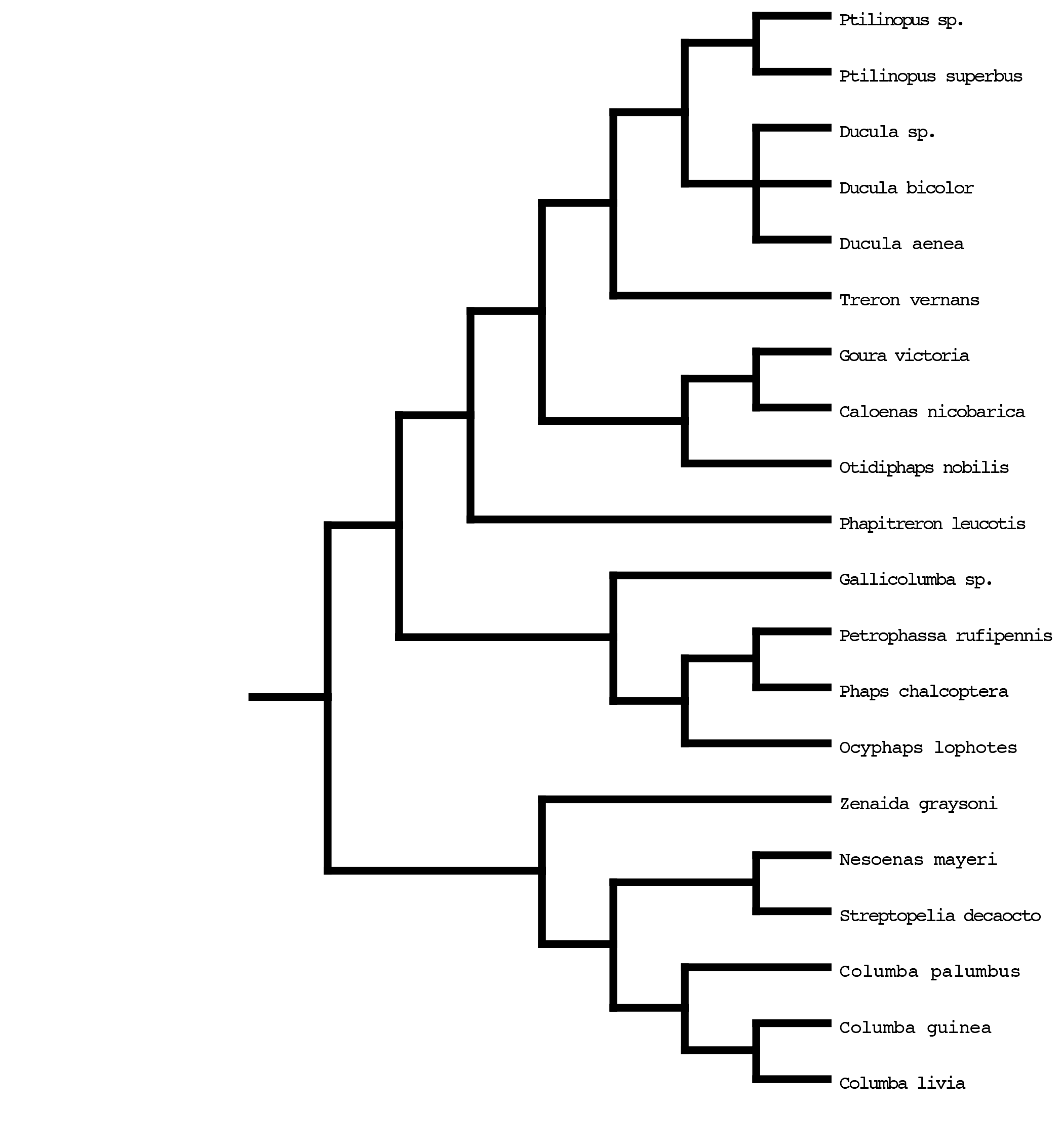


**Supplementary Material S3.** Raw convex hull volume data for dodo dataset. Volume in mm^3^.

|  | **Trunk** | **Skull** | **Neck** | **RFem** | **RTbt** | **RTmt** | **RFoot** | **RHum** | **RRad** | **Rcmc** |
| --- | --- | --- | --- | --- | --- | --- | --- | --- | --- | --- |
| NHMUK Kensington | 6953891 | 765172 | 434765 | 158901 | 321590 | 42267 | 317967 | 57545 | 14204 | * |
| NHMUK Tring | 6030985 | 839790 | 291411 | 178101 | 336612 | 50967 | 248958 | 51480 | 15910 | 6399 |
| Edinburgh | 9671000 | 788000 | 329000 | 140000 | 259000 | 72000 | 167000 | 24000 | 20000 | 8000 |

|  | **LFem** | **LTbt** | **LTmt** | **LFoot** | **LHum** | **LRad** | **Lcmc** |
| --- | --- | --- | --- | --- | --- | --- | --- |
| NHMUK Kensington | 142920 | 268549 | 52379 | 128606 | 56471 | 15141 | * |
| NHMUK Tring | 163822 | 352163 | 52348 | 248729 | 49864 | 17840 | 7441 |
| Edinburgh | 124000 | 237000 | 77000 | 193000 | 20000 | 14000 | 4000 |

*carpals not present in the NHMUK Kensington mount
